# Early resection of a rare congenital pulmonary airway malformation causing severe progressive respiratory distress in a preterm neonate: a case report and review of the literature

**DOI:** 10.1186/s12887-023-04049-3

**Published:** 2023-05-13

**Authors:** Megan Ottomeyer, Charles Huddleston, Rachel M. Berkovich, David S. Brink, Joyce M. Koenig, Kurtis T. Sobush

**Affiliations:** 1 Department of Pediatrics, Division of Neonatology, Saint Louis University, St. Louis, MO 63104 USA; 2 Department of Surgery, Division of Pediatric Cardiothoracic Surgery, Saint Louis University, St. Louis, MO 63104 USA; 3 Department of Radiology, SSM Health Cardinal Glennon Children’s Hospital, St. Louis, MO 63104 USA; 4 Department of Pathology, Division of Pediatric Pathology, Saint Louis University, St. Louis, MO 63104 USA; 5 Department of Molecular Microbiology & Immunology, Saint Louis University, St. Louis, MO 63104 USA; 6 Department of Pediatrics, Division of Pediatric Pulmonary and Sleep Medicine, Saint Louis University, St. Louis, MO 63104 USA

**Keywords:** CPAM, Congenital lung malformation, Prematurity, Respiratory distress

## Abstract

**Background:**

Congenital pulmonary airway malformations (CPAMs) are a heterogenous collection of congenital lung malformations, often diagnosed prenatally. The Stocker Type III CPAM is a rare CPAM sub-type, and, when large, may be associated with hydrops. Furthermore, reports of CPAM management which may include surgical resection in extreme preterm infants are limited.

**Case presentation:**

We report a case of a female neonate born at 28 weeks of gestation with severe respiratory distress and diffuse pulmonary opacification on the right concerning for a large congenital lung lesion. This lesion was not detected on routine antenatal imaging, and she did not have clinical findings of associated hydrops. Her respiratory status improved dramatically after surgical resection of a mass at 12 day of age. The mass was consistent pathologically with a Stocker Type III CPAM. Lung expansion showed subsequent improvement at 16 months of age.

**Conclusions:**

Our case describes a preterm neonate with severe respiratory distress that was found postnatally to have a large, unilateral congenital lung lesion despite a normal prenatal ultrasound. Additionally, this lesion required excision early in life due to severity of respiratory compromise. This case highlights that rare congenital lung lesions, like this rare sub-type of CPAM, should remain a diagnostic consideration in neonates with severe respiratory distress. Early lung resection for CPAM in preterm infants is not well described and the favorable outcomes of this case help expand perspectives on potential management strategies.

## Background

Congenital lung malformations are a group of congenital anomalies, with an estimated prevalence of about 30–42 cases per 100,000 individuals [1] or 1 in 10,000 live births [2]. Congenital lung malformations overall are frequently diagnosed prenatally, with about 60% of cases diagnosed in the fetal period in one multi-center registry [3]. The most common congenital lung lesions are congenital pulmonary airway malformations (CPAMs), formerly known as congenital cystic adenomatoid malformations (CCAMs), [4] with a prevalence previously estimated to be 1 per 25,000–35,000 live births [2]; with recent improvements in quality of prenatal ultrasound, the prevalence of CPAM is estimated to have increased three-fold in the years 1997 to 2009 [3].

In general, CPAMs, as well as other congenital lung malformations, are isolated anomalies that are diagnosed on routine prenatal ultrasound in up to 70% of cases [5], typically between 18 and 20 weeks gestational age, but are often difficult to characterize fully in the fetal period [4, 6–9]. Fetal magnetic resonance imaging (MRI) has shown promise as a prenatal imaging modality frequently consistent with postnatal pathological diagnosis [2, 10]. Other associated findings prenatally can include polyhydramnios or hydrops secondary to mass effect associated with the CPAM leading to compression of the fetal esophagus or inferior vena cava [1, 4, 8, 11, 12].

We report a case of a 28- week gestational age female neonate with a normal antenatal ultrasound with respiratory distress at birth, found postnatally to have a large right-sided solid lung mass. Due to escalation of respiratory clinical symptoms, surgical intervention was expedited in this case relative to a typical timeline even with little being known on the impact of CPAM removal in preterm infants in the literature. This case demonstrates a rare sub-type of CPAM presenting as severe respiratory distress in a preterm neonate, which atypically was not diagnosed on routine prenatal imaging and required surgical resection in the first month of life due to the severity of respiratory compromise.

## Case presentation

A female infant was delivered by spontaneous, vaginal delivery after preterm labor at an outlying referring hospital at 28 weeks’ gestation. The infant’s mother, a 26-year-old primigravid woman with a family history negative for congenital malformations and positive for Factor VII deficiency, presented to the referring facility in preterm labor. She had received routine prenatal care; polyhydramnios and concerns for placenta previa were noted as complications to her prenatal course by antenatal ultrasound at 21 weeks gestation though no fetal organ anomalies were reported. She received magnesium sulfate, fentanyl, and a single dose of betamethasone prior to delivery. Antibiotics were not administered prior to delivery given the short duration between presentation and delivery. A bedside ultrasound of the mother on day of delivery revealed a low-lying placenta without previa, as well as polyhydramnios with deepest vertical pocket at 13.98 cm.

Following delivery, the infant was bradycardic and without respiratory effort. She received bag-mask ventilation and chest compressions for bradycardia for the first 2 min of life. After resolution of bradycardia, chest compressions were stopped, and bag-mask ventilation was continued. High inspiratory pressures were required with bag-mask breath delivery to attain acceptable chest rise and ventilation. The infant also received a 10 mL/kg saline infusion in the delivery room. The infant was intubated and placed on synchronized intermittent mandatory ventilation (SIMV) with pressure-targeted breaths. She received endotracheal surfactant and budesonide, per institutional protocol for very low birthweight (VLBW) infants, which were both well-tolerated. A blood culture was obtained with ampicillin and gentamicin therapy subsequently being initiated. Despite maximal (FiO2 100%) oxygen therapy, oxygen saturations remained in the 70–80% range. Initial chest radiograph at the referring facility was concerning for complete opacification of the right lung field.

**Fig. 1:**
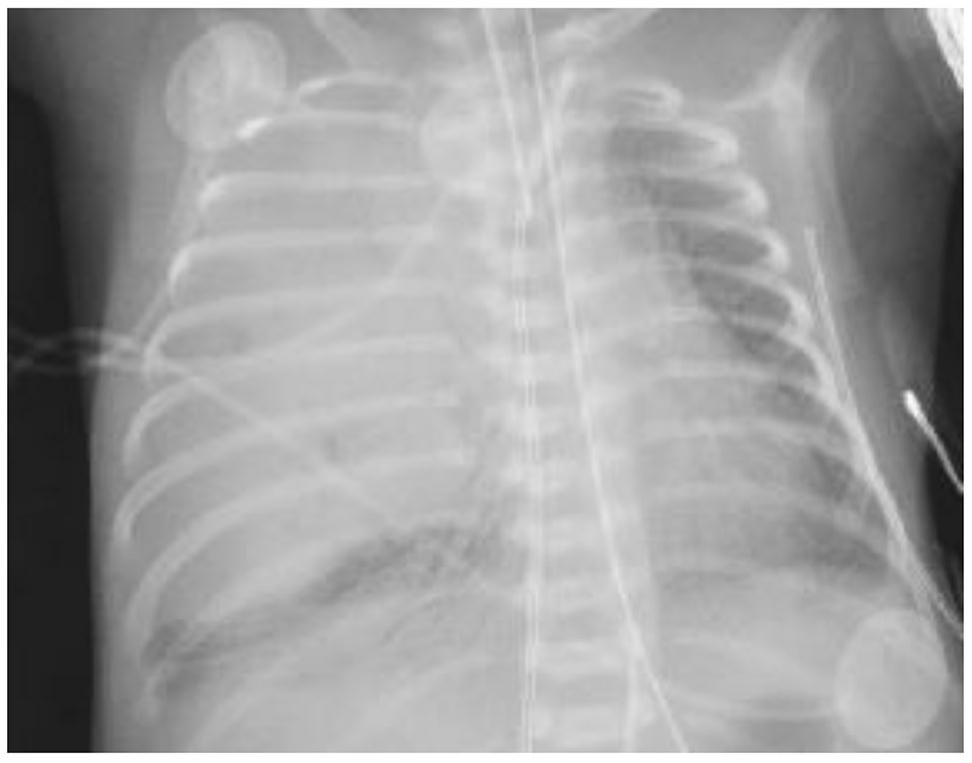
Anterior-Posterior Chest X-Ray at 5 h of Age, Right Lung Field Homogenous Opacification

The infant was transferred at 5 h of life by air transportation to a level IV neonatal intensive care facility. On her arrival, she was placed on high-frequency-oscillatory ventilation (HFOV), with rapid improvement in oxygen saturations (> 90%) while on 100% FiO2. Chest radiograph upon arrival demonstrated a predominantly homogeneous opacification of the right lung field with scant bronchial airway markings and a left-ward mediastinal shift (Fig. 1). These findings prompted concern for a possible lung mass and led to the performance of a chest ultrasound which showed a large region of heterogeneous hyperechogenicity within the right chest and scattered irregular cystic-appearing spaces. An irregular branching vascular structure was seen with color doppler flow imaging adjacent to the cystic spaces without definitive connection to the aorta. Color doppler flow imaging also revealed other regions of internal vascularity within the mass. Atelectatic lung was also observed subjacent to the mass in the right lung base. A subsequent contrast-enhanced computerized tomography (CT) scan of the chest was recommended.

**Fig. 2:**
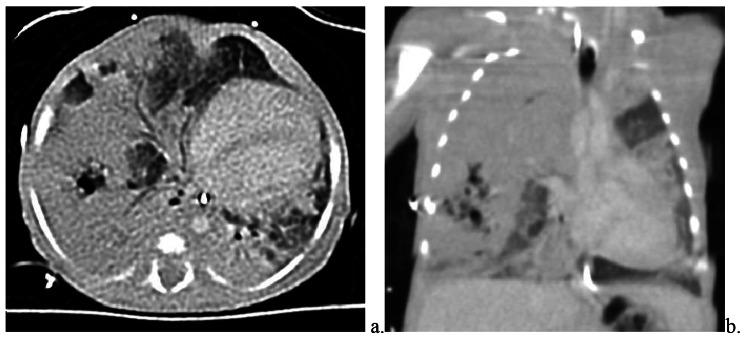
Computed Tomography (CT) Scan with Contrast, Chest. Transverse (**a**) and Coronal (**b**) Views

Difficulties with oxygenation continued, prompting addition of inhaled nitric oxide (20 ppm) to the HFOV ventilatory regimen. Echocardiogram at that time demonstrated elevated right ventricular pressures based on intraventricular septum flattening as well as mild right ventricular dilation and low-normal right ventricular systolic function. Left ventricular systolic function was normal. A patent foramen ovale with bidirectional shunting as well as a moderate patent ductus arteriosus with left-to-right shunting were seen, but the heart was otherwise structurally normal. A dopamine infusion as well as stress-dose hydrocortisone were instituted for the treatment of hypotension. Over the course of one week, the respiratory status began to gradually deteriorate despite continuation of maximal ventilator support. The infant was successfully, though briefly, converted to SIMV in order to obtain the previously recommended CT scan of the chest to better define the lung lesion and guide management. Chest CT with contrast (Fig. 2) revealed a large area of a relatively homogeneous soft tissue density involving the majority of the right hemithorax with sparing of the right inferior hemithorax and extension across midline with left lateral displacement of the anterior junction line. Notably, a focus of loculated air density with irregular margins was noted in this area and measured 1.9 cm x 1.3 cm x 1.9 cm. A normal distribution of bronchi and pulmonary vascular structures was seen coursing throughout this area of opacification. No systemic vessels were observed to supply the mass that would raise concern for sequestration. Apparent effacement of a long segment of the bronchus to the right upper lobe with suspicion of partial effacement of the bronchus to the lateral segment of the right middle lobe were also observed.

**Fig. 3:**
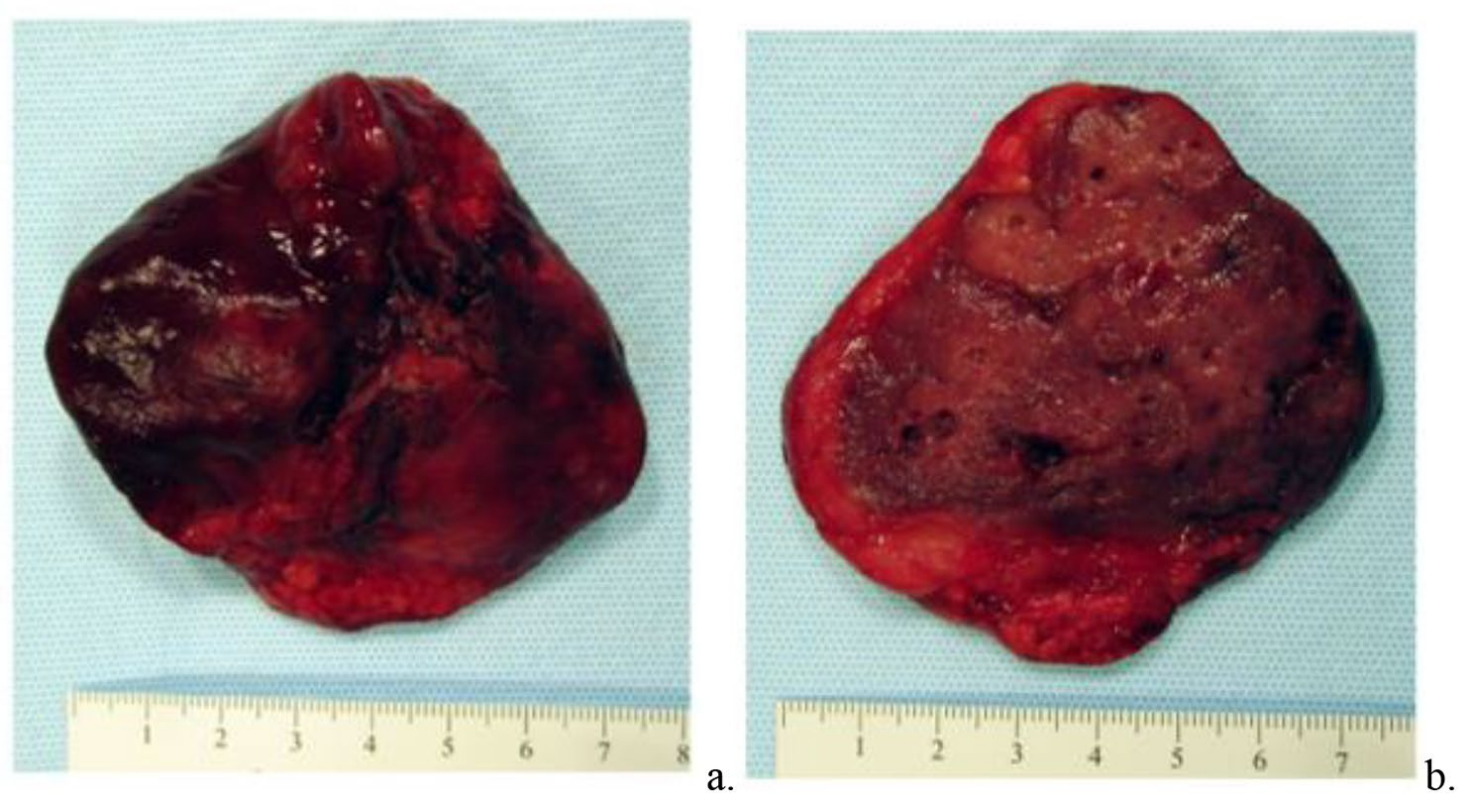
Gross Pathologic Specimen of Resected Right Upper Lobe Mass, External (**a**) and Cut Surface (**b**). The cut surface shows the lesion to be spongy and dark red with adjacent, paler normal lung parenchyma

**Fig. 4:**
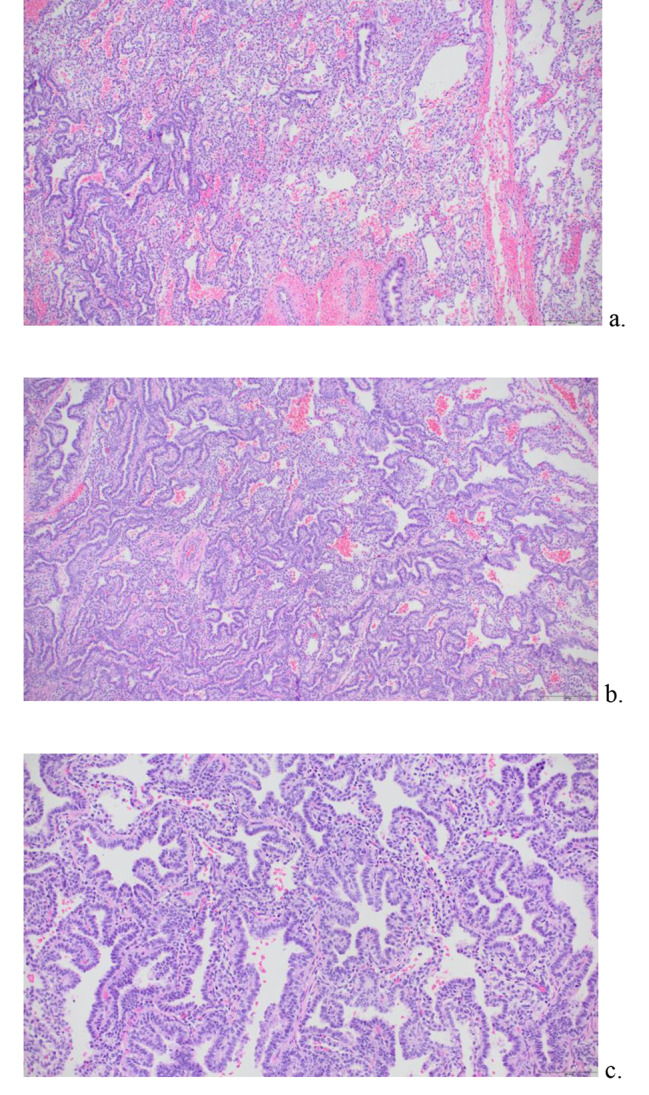
Histology of Resected Right Upper Lobe Mass, Low Magnification. Histologically (**a-c**), the lesion shows stellate spaces imparting an adenomatoid appearance. In Fig. 4a, the unencapsulated lesion (left) merges with normal lung (right)

**Fig. 5:**
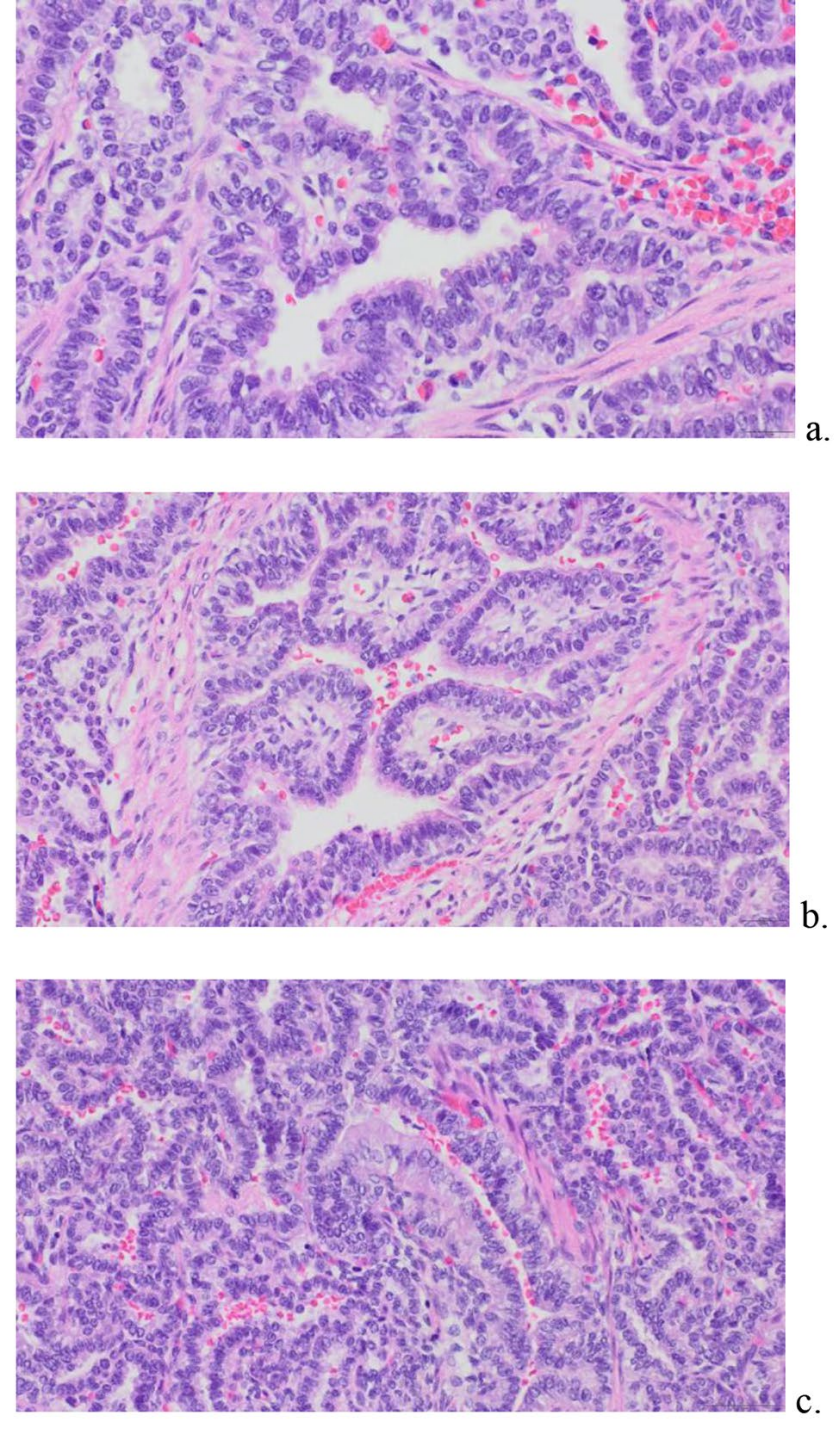
Histology of Lung Lesion at High Magnification. Histology of Resected Right Upper Lobe Mass, Higher Magnification. Higher magnification shows the stellate spaces to be lined by cuboidal to low columnar cells (**a, b**) and rare mucous cells (**c**)

Due to the persistent mass occupying the majority of the right hemithorax and the mass effect on adjacent bronchi and ipsilateral lung, the infant’s respiratory status became increasingly labile and increased inotropic support was needed for hypotension. The infant’s size precluded use of additional supportive measures such as extracorporeal membrane oxygenation (ECMO). Given worsening of the infant’s respiratory and circulatory status and after obtaining consent, on day 12 of life a bedside open thoracotomy for excision of the lesion was performed in joint effort between the pediatric cardiothoracic surgery and pediatric surgery teams. This was performed without an initial surgical attempt at a diagnostic biopsy because of the perceived escalating need for therapeutic removal. Following right posterolateral thoracotomy at the 5th intercostal space, it was visually apparent that the right upper lobe was completely noncompliant while bilateral lungs were receiving mechanical ventilation. Additionally, the right middle and lower lung lobes appeared normal with adequate compliance after the right upper lobe was elevated to expose the hilum. After ligation of both the right upper lobe pulmonary vasculature and the effaced right upper lobe bronchus (accomplished while the right lung was being mechanically ventilated), a 7.5 × 6 × 2.6 cm, 57 gram lesion of the right upper lobe was resected and sent for pathological examination (Figs. 3, 4 and 5). At the time of removal of the mass, the tissue of the right upper lobe of the lung was described as having consistency of consolidated tissue similar to the liver (Fig. 3). The margins of the lesion were demarcated grossly compared to the surrounding normal appearing lung and were limited to the right upper lobe. Pathologic analysis showed the lesion to be unencapsulated with an adenomatoid appearance merging with adjacent lung parenchyma (Fig. 4). Histopathologic analysis revealed the mass was also characterized by irregularly shaped spaces lined with ciliated low-columnar to cuboidal epithelium overlying an incomplete layer of mature smooth muscle (Fig. 5). These findings were characteristic of type III CPAM.

**Fig. 6:**
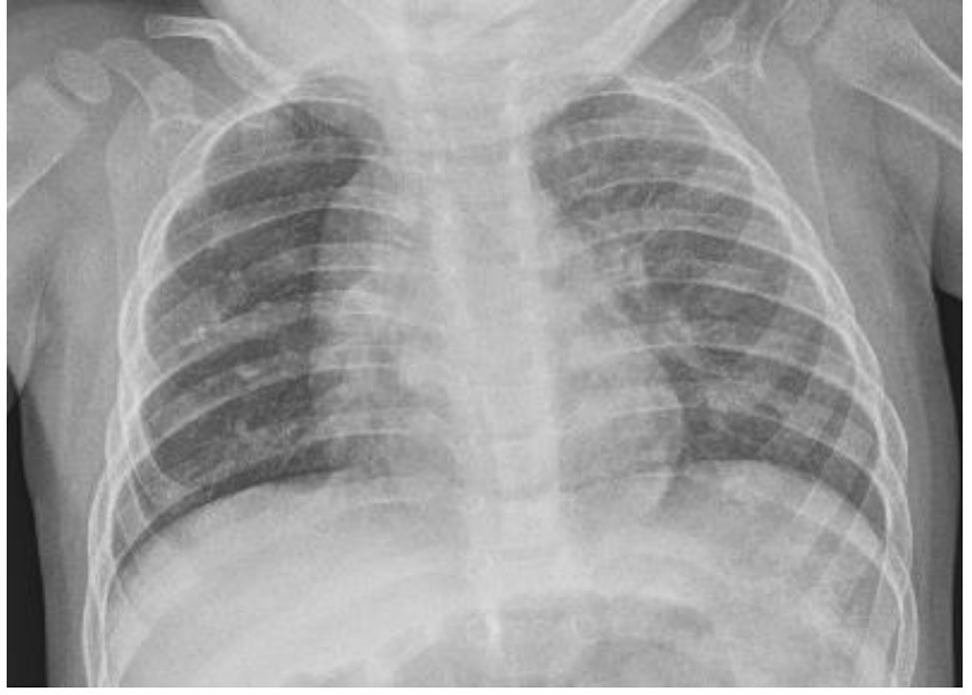
Postoperative Chest X-Ray Demonstrating Right Lung Expansion at 16 Months of Age

After removal of the mass, the previously compressed right middle and lower lobes were observed to immediately inflate, and a significant decrease in ventilator settings was required shortly thereafter. During the initial postoperative recovery, the infant’s respiratory status continued to dramatically improve. Within 48 h, she was successfully extubated to bubble CPAP, and she subsequently transitioned to oxygen by nasal cannula. She was discharged home on 1/8 L/min oxygen via nasal cannula at 3 months of life. The infant did well at home, requiring supplemental oxygen ranging from 1/8 L/min to 1/2 L/min, which was discontinued completely at 4 months of age. She continues to be followed by a pediatric pulmonologist and currently exhibits normal room air oxygen saturations. Of note, infant pulmonary function testing was not pursued for this patient as it was not performed at the treating intensive care facility while the improvement in work of breathing and oxygen status justified limiting pursuit at an outside institution. There have not been recurrent lung infections nor any clinical evidence of lower airway obstruction warranting additional medical treatment. A follow-up chest x-ray obtained at 16 months of age was consistent with normal lung fields and normal mediastinal position (Fig. 6). Throughout her course, the infant had normal head ultrasounds at day 7 of life as well as at term corrected age (following surgical intervention) and did not demonstrate significant neurodevelopmental deficits at long-term follow up visits.

## Discussion and conclusions

Our patient was diagnosed with CPAM type III after premature birth at 28 weeks of gestation. This lesion resulted in significant respiratory difficulties with both ventilation and oxygenation. The infant tolerated surgical resection of the lung lesion at 12 days of age and had a relatively unremarkable postoperative course, requiring only minimal oxygen support at hospital discharge. She is currently thriving at home, on room air with normal imaging studies.

A discussion of CPAMs, including their etiology, natural history, diagnosis, and outcomes, follows.

***Etiology of CPAMs.*** The etiology of CPAMs involves disruption of the normal airway patterning during the course of lung development [4, 9] most likely occurring in the pseudo-glandular stage [11, 13]. During lung morphogenesis, which begins as early as 3 weeks’ gestation and continues postnatally, airway branching involves extensive signaling crosstalk between respiratory epithelium and the surrounding mesenchyme [13]. Several factors involved in this process have been shown to play a possible role in the development of CPAM. These mediators include the following: fibroblast growth factor (FGF)-7, 9 and 10; thyroid transcription factor 1 [4, 11]; bone morphogenic protein (BMP)-4; platelet-derived growth factor (PDGF)-B; sonic hedgehog (SHH); glial cell-derived neurotrophic factor; and sex-determining factor Y-box-2 gene (SOX2) [4, 6, 14]. Development of CPAM may also be influenced by the development of concomitant anomalies of large airways, such as bronchial atresia or in utero airway obstruction [15] similarly to what has been described in other congenital lung malformations, such as bronchogenic cysts [12].

***Radiographic findings of CPAMs.*** When present, CPAM is commonly diagnosed on prenatal ultrasound between 18 and 20 weeks of gestation [4, 6–9]. Prenatal ultrasound findings typically include a heterogenous mass with a single or multiple hyperechoic cystic structure(s) [4, 11, 16]. The dimensions of these cysts are highly variable; they may include microcystic structures that may appear as a solid anechogenic mass [4, 16]. While prenatal ultrasound is a diagnostic imaging modality with a sensitivity approaching 90% for detection of CPAM, findings seen on prenatal ultrasound are not always predictive of histopathological diagnosis, yielding a specificity of approximately 75% [4, 10]. Additionally, the sensitivity of prenatal ultrasound in the detection of cystic lung lesions decreases as pregnancy progresses due to changes in fluid-tissue interface [11]. After diagnosis on prenatal imaging, these lesions are typically followed closely with serial ultrasounds; fetal MRI may further characterize their anatomy [2, 4, 10, 16], typically as a T2 hyperintense mass [16]. Fetal MRI has shown recent promise as a more specific prenatal diagnostic imaging modality compared to prenatal ultrasound. Fetal MRI showed a high (83%) agreement with pathologic diagnosis [2, 10]. This represents improvement over the diagnostic accuracy of findings on prenatal ultrasound (67% agreement with pathologic diagnosis) [10].

***Histologic, clinical, and radiographic findings of CPAM sub-types.*** The radiographic, clinical, and pathological characteristics of CPAMs are highly variable. In the case series by Stocker et al. in 1977, a combination of clinical and histopathological features were used to categorize CPAMs into three subtypes (Types I-III) [17], with two additional subtypes (Types 0 and IV) added more recently [18]. The significant overlap between lesion subtypes as well as the difficulty in classifying atypical lesions have led to the proposal of additional classifications of CPAMs, such as division of CPAMs into large-cyst type or small-cyst type as proposed [15] or categorization based on cell type via immunohistochemistry [19].

Among CPAMs, Stocker Type I lesions are the most common, representing 60–70% [11, 20]. They are characterized by a large single cyst (> 10 cm) or multiple small cysts (2–10 cm) in a multi-loculated mass affecting a single lobe [17]. Cysts are typically lined by pseudostratified columnar epithelium, with or without “islands” of mucigenic epithelium [21]. This subtype is consistent with the large-cyst type of CPAM described by Langston [15] and consistent with the bronchiolar epithelium type described by Morotti [19]. Notably, Stocker Type I CPAMs are associated with progression to bronchioalveolar carcinoma in about 1% of cases [12].

Stocker Type II CPAMs are sponge-like masses composed of a group of uniform, evenly spaced, small (0.5-2 cm) cysts, affecting part or all of a single lobe [11, 17]. These CPAMs are equivalent to the small-cyst type of CPAM described by Langston [15]. The frequent presence of normal-appearing intervening lung parenchyma can make clear delineation of these lesion difficult. Cysts are lined with ciliated cuboidal to columnar epithelium [21] and are immunohistochemically consistent with bronchiolar epithelium [19].

Stocker Type III CPAMs are rare, accounting for only 5–10% of CPAMs [11, 20]. These bulky, solid masses may or may not include cystic structures, with cysts measuring less than 0.2 cm. [17]. Histologically, lung tissue appears inappropriately immature for gestational age with excessive growth of terminal airspaces. If cysts are present, they are often lined by ciliated cuboidal epithelium [11, 21] and are immunohistochemically consistent with bronchiolar epithelium [19]. This subtype can affect a single lobe or the entire lung and is most likely to be associated with mediastinal shift [21].

Clinically, CPAMs of all types have a wide range of presentations. CPAMs are often diagnosed only as an incidental radiographic finding in asymptomatic children in 40–60% of cases [7, 22]. CPAMs may also clinically present as respiratory distress, pneumothorax, recurrent pneumonias, or wheezing [3–5, 7, 11, 22].

Radiographically, Stocker Types I and II likely appear as hyperlucent cystic areas on chest x-ray and CT, whereas Stocker Type III CPAMs typically appear as radio-opaque solid masses, though small areas of hypoattenuation may be seen based on if the lesion has a microcystic component [16]. Interestingly, in our case, CT imaging revealed an area of air density measuring larger than 0.2 cm within the solid lesion, which would not be consistent with designation as a Stocker Type III CPAM, despite this being the final pathologic diagnosis based on histologic findings.

Stocker Type IV and Type 0 CPAMs are less common subtypes. Stocker Type IV CPAMs are masses of large air-filled alveolar cysts, located peripherally and involving only a single lobe [18]. The cysts are thin-walled and lined by simple squamous or cuboidal epithelium [21] and, immunohistochemically, are consistent with acinar-alveolar epithelial cell differentiation [19]. This subtype accounts for less than 5% of CPAMs [16, 20] and may be a well-differentiated form of cystic pleuropulmonary blastoma (PPB) [21]. In fact, radiographically, these lesions are indistinguishable from PPB [16]. Stocker Type 0 CPAMs account for 1% of CPAMs [11]. These lesions are associated with acinar dysplasia, leading to severe pulmonary hypoplasia and, if affecting both lungs, are incompatible with life [21].

The radiographic findings in our case favored diagnosis of fetal lung interstitial tumor (FLIT) given the solid nature of the lung lesion on imaging and the fact that a lesion this large was not detected on prenatal imaging, with most recent prenatal ultrasound done at 21 weeks gestation. Stocker Type III CPAM was also on the differential; however, the large area of air density measuring 1.9 cm x 1.3 cm by 1.9 cm seen within the mass made this diagnosis less likely. Given the severity of illness, a postnatal MRI was not done; rather, the lesion was surgically excised. After excision, the pathologic diagnosis was more consistent with Stocker Type III CPAM, given the ciliated low columnar to cuboidal appearance of epithelium and lack of a fibrous capsule. This underscores the many overlapping characteristics of various congenital lung lesions radiologically, and the need for histological examination for definitive diagnosis.

***Natural history of CPAMs***. The natural history and progression of CPAMs have been variously described among studies. Up to 44% of CPAMs may increase in size during pregnancy [4, 11]. The growth velocity typically peaks between 20 and 26 weeks’ gestation [11], with continued growth being rare after 28 weeks. In contrast, an estimated 8–42% of CPAMs regress in size during fetal life [4, 8], and 11–49% of CPAMs completely resolve before birth [4]. The severity of clinical presentation of CPAMs in the neonatal period also varies widely, from being completely asymptomatic in up to 70% of neonates [4], to more severe problems that include respiratory distress, pulmonary hypertension, and complications of hydrops among nearly all sub-types of CPAM [4, 7, 10–12].

***CPAM and prognosis.*** The presence of hydrops is the most significant prognostic factor for prenatally diagnosed CPAMs [1, 4, 7, 12, 20, 22, 23]. One tool used to predict a potential for associated complications is the CPAM volume ratio (CVR). The CVR is calculated by multiplying the CPAM volume (length x width x height) by a constant (0.52), which is then divided by the estimated head circumference to normalize for gestational age [23]. A CVR greater than 1.6 is highly predictive of hydrops [23]. More recent studies suggest that a CVR greater than 0.84 is highly predictive of severe respiratory distress at birth, particularly in the context of polyhydramnios and ascites [4]. A CVR less than 1.6 may also be associated with hydrops, but only in the presence of a dominant cyst [23].

In our patient, the CPAM was postnatally calculated to have a CVR of 2.21, which would be highly predictive of hydrops. However, our patient had no clinical evidence of hydrops though there was prenatal evidence of polyhydramnios.

The variability in both the expected prenatal course as well as postnatal presentation of CPAM makes it difficult to accurately predict outcomes and provide appropriate prenatal management. Prenatal management options for CPAMs have focused primarily on those lesions most associated with complications, such as hydrops or pulmonary hypertension. Antenatal maternal steroid administration has demonstrated some success in reversing CPAM-associated hydrops [4, 11, 12, 24], particularly with microcystic CPAM lesions. Additionally, antenatal steroids have lessened the risk of premature birth and mechanical ventilation requirements in CPAM compared to fetal surgical management [24]. Other prenatal interventions include percutaneous ultrasound-guided sclerotherapy, percutaneous cystic aspiration, thoraco-amniotic shunting [11, 12], and fetal lobectomy/resection [4, 24]. Additionally, ex utero intrapartum treatment (EXIT) interventions hold promise for infants specifically with large lesions causing mediastinal shift and the decreased ability to survive until term [4, 11].

The infant in our case was born at a gestational age of 28 weeks, making it unlikely that her CPAM would have continued to enlarge should the pregnancy have continued until term. Conversely, there is a high likelihood that the mass would have decreased in size, possibly even resolving completely. However, given the high estimated CPAM volume ratio (CVR) and its close association to hydrops, it is also possible that a continuation of this pregnancy to term may have resulted in clinically significant hydrops (such as effusion or ascites) and its sequelae.

***Postnatal management of CPAM.*** Initial steps in postnatal management of CPAMs often involve further characterization of the mass, as this is often not reliable on prenatal imaging. Postnatal CT remains the gold standard for confirming diagnosis, with varied but overall good concordance of radiographic diagnosis and histopathologic diagnosis in multiple studies [4, 9, 10]. Additionally, CT with contrast allows for the definitive delineation of vascular supply to the lesion and whether that supply originates from the pulmonary or the systemic circulation [4, 10, 16]. This may be able to be seen on prenatal ultrasound with doppler color flow interrogation [8, 11, 16], though with sensitivity as low as 49%, [10] or fetal MRI [2]. CT scan to further characterize prenatally diagnosed congenital lung masses in asymptomatic infants is recommended within the first 3 [11, 12] to 6 months of life [4], and sooner if symptomatic as seen with this case. However, postnatal CT was shown to be redundant in cases where fetal MRI was performed and a systemic feeding vessel supplying the mass was detected [2]. The presence or absence of this systemic feeding vessel is an important distinction, as it is an important diagnostic criterion for other congenital lung malformations, such as bronchopulmonary sequestrations or hybrid lesions [2, 3, 9, 10, 15, 16, 21, 22].

The accepted treatment of symptomatic CPAM is surgical resection [1, 11, 12, 20, 22, 24, 25]. Resection is generally recommended within the first year of life in order to optimize compensatory lung growth [1, 4, 11, 20, 22, 25], with a reported median age of resection at 5 months in a retrospective review over a 10-year period at Oxford as reported by Thakkar [25]. However, one study showed no differences in spirometry results at age 10 years in children with resection of CPAM prior to 2 years of age or after 2 years of age, and infants with resected CPAMs generally have good long-term outcomes with normal lung function and growth [11]. In contrast, the postnatal management of asymptomatic CPAM remains controversial [4, 11, 12, 15, 22]. Asymptomatic infants with CPAM managed conservatively typically develop symptoms including cough, wheezing, and recurrent pneumonia within the first 6 months of life [11, 12], and presence of these symptoms has been associated with increased likelihood of post-operative complications and prolonged hospitalization after resection [12].

***Differential diagnosis of congenital lung masses.*** The most common congenital lung malformation is the CPAM lesion. However, the differential diagnoses of congenital lung masses include the following: pulmonary sequestration, bronchogenic cyst, congenital lobar emphysema, [4, 9, 10, 12, 21] and FLIT [26].

Intra-lobar pulmonary sequestrations or “hybrid lesions” are masses of lung tissue within the visceral pleura with histological characteristics consistent with Stocker Type II CPAM but with the presence of an anomalous feeding vessel arising from the systemic, rather than pulmonary, circulation [21]. Extra-lobar pulmonary sequestrations may have histological features of Stocker Type II CPAM as well as anomalous arterial supply from the systemic circulation. However, unlike intra-lobar pulmonary sequestrations, these lesions are invested in their own pleura. Both intra-lobar and extra-lobar pulmonary sequestrations generally appear as radio-opaque masses on postnatal chest x-ray. A systemic feeding vessel is typically confirmed by postnatal CT [2, 8, 16], although these anomalous vessels can be seen on prenatal ultrasound and prenatal MRI [8, 16]. Intra-lobar pulmonary sequestrations are often asymptomatic at birth. In contrast, extra-lobar pulmonary sequestrations have been associated with neonatal respiratory distress, and large lesions may be accompanied by hydrops [22].

Bronchogenic cysts are unilocular cysts arising from the bronchopulmonary foregut and are most commonly found in the mediastinum [21]. They do not communicate with the tracheo-bronchial tree and are lined by pseudostratified columnar respiratory epithelium with cartilage present in the cyst wall [20, 21]. The cysts are often fluid-filled, resulting in radio-opaque appearance on chest x-ray, or as a low-attenuating mass on CT. Post-natal MRI can reveal hyperintense T2 signaling with variable T1 signal [16].

FLITs are well-circumscribed intraparenchymal lung masses first described by Dishop in 2010 [26]. These solid masses are composed of immature airspaces and interstitial tissue that resemble lung tissue in the canalicular stage of development. However, these lesions are abruptly separated from normal lung by a fibrous border. Histologically, low cuboidal epithelial cells are present with polygonal mesenchymal cells [26]. Similarly to CPAMs, these lesions have been associated with hydrops secondary to impairment of venous return and/or esophageal compression [27].

***CPAM surgical management in preterm infants.*** Reports of preterm infants undergoing surgical resection of CPAM are limited and variable. Two different reports of a case series describe surgical resection in the context of known prenatal ultrasound findings of a CPAM with emergency cesarean delivery and immediate surgical intervention on the first day of life [28, 29]. In these cases, maternal steroids were given with minimal improvement seen in CVR. Polyhydramnios was reported in almost all cases in those that required urgent surgery in the early preterm period. Mediastinal shift with notable displacement of the heart often accompanied the CPAM lesions where surgical resection was pursued. Reports are seen of favorable outcome from postnatal surgical resection of CPAM lesions between 27 and 33 weeks gestation however it is important to note that case of intrauterine fetal demise are excluded from these types of studies even though there could be pathologic similarities in CPAM lesions seen.

The heterogenous group of congenital lung malformations known at CPAMs present with highly variable clinical, radiographic, and histological features. While CPAMs are most commonly diagnosed prenatally, our present case shows that a large, clinically significant CPAM may not be detected by routine antenatal imaging, which can complicate anticipatory care. Our case also describes a relatively rare CPAM sub-type leading to severe respiratory distress but not associated with hydrops despite an elevated CVR. The degree of respiratory distress necessitating resection of the lesion in the first days of life, as presented in our case, is also unusual for CPAMs. Few cases are reported in the literature regarding outcomes of infants requiring resection of CPAMs within the first days of life, and to our knowledge, none of these cases involved an extremely premature infant. Additionally, our case demonstrates variation among typical radiographic findings of Stocker Type III CPAMS, expanding the already diverse array of clinical presentations and diagnostic findings associated with CPAMs.

## Data Availability

Data sharing is not applicable to this article as no datasets were generated or analyzed during the current study.

## Abbreviations

BMP: Bone morphogenic protein
CCAM: Congenital cystic adenomatoid malformation
CPAM: Congenital pulmonary airway malformation
CT: Computed tomography
CVR: CPAM volume ratio
ECMO: Extracorporeal membrane oxygenation
EXIT: Ex utero intrapartum treatment
FGF: Fibroblast growth factor
FiO2: Fraction of inspired oxygen
FLIT: Fetal lung interstitial tumor
HFOV: High-frequency-oscillatory ventilation
MRI: Magnetic resonance imaging
PDGF: Platelet-derived growth factor
PPB: Pleuropulmonary blastoma
PPM: Parts per million
SHH: Sonic hedgehog
SIMV: Synchronized intermittent mandatory ventilation
SOX2: Sex-determining factor Y-box-2
VLBW: Very low birthweight
